# Understanding the Tripartite Relationship Between Dietary Practices, Psychological Well-Being, and Disease Experience in Greek Patients with IBD: A Mixed-Methods Exploration

**DOI:** 10.3390/nu18091439

**Published:** 2026-04-30

**Authors:** Dimitra Eleftheria Strongylou, Vaios Svolos, Athanasia Vlachou, Elli Zoupa, Vasiliki-Rafaela Vakouftsi, Anastasia Ntanou, Konstantinos Argyriou, Andreas Kapsoritakis, Fotini Bonoti, Odysseas Androutsos

**Affiliations:** 1 Lab of Clinical Nutrition and Dietetics, Department of Nutrition and Dietetics, School of Physical Education, Sports Science and Dietetics, University of Thessaly, 42100 Trikala, Greece; ntemi_st@hotmail.com (D.E.S.); vaiossvolos@gmail.com (V.S.); nasiavlachou@outlook.com (A.V.); zoupaelli@gmail.com (E.Z.); fbonoti@uth.gr (F.B.); 2 School of Medicine, Dentistry and Nursing, College of Medical, Veterinary and Life Sciences, University of Glasgow, Glasgow G12 8QQ, UK; 3 Hellenic Society of Crohn’s Disease’s and Ulcerative Colitis’ Patients (HELLESCC), 11252 Athens, Greece; vasovak@yahoo.com (V.-R.V.); nastiantanou@hotmail.com (A.N.); 4 Department of Gastroenterology, University Hospital of Larisa, 41100 Larisa, Greece; koargyri@med.uth.gr (K.A.); kapsoritakis@uth.gr (A.K.)

**Keywords:** Crohn’s disease, ulcerative colitis, inflammatory bowel disease, mental health, qualitative research, diet, nutrition, experiences, beliefs, disordered eating

## Abstract

**Background/Objectives**: Diet and mental health constitute two significant modifiable factors affecting Inflammatory Bowel Disease (IBD). The present exploratory study explores potential interrelationships between mental health and eating patterns in IBD patients in Greece. **Methods**: A mixed-methods approach was followed. Two hundred and eighty-three individuals living with IBD in Greece (*n* = 110 UC, *n* = 173 CD) participated in an online questionnaire survey examining demographic characteristics, anxiety, depression, and dietary attitudes. Fourteen semi-structured interviews explored the lived experiences of diet, mental health, and disease among IBD patients. **Results**: Quantitative study revealed that 45.77% of patients scored above the clinical cutoff for anxiety (GAD-7 ≥ 10) and 48.37% for depression (PHQ-9 ≥ 10). Patients with UC exhibited a significantly higher prevalence of moderate to severe anxiety (54.5% vs. 37.0%, *p* = 0.004) and depression (54.5% vs. 42.2%, *p* = 0.042) compared to CD. Disordered eating attitudes were present in 27.22% of the total sample, with no significant differences between diagnostic groups (*p* = 0.985). Thematic analysis revealed three overarching themes, namely (a) ‘life in two phases: IBD impact on health’, (b) ‘mental health and diet interplay—their perceived impact on IBD’ and (c) ‘coping strategies for managing IBD’. **Conclusions**: Our findings highlight the dynamic interplay among diet, mental health, and IBD experience. The study underscores the importance of developing holistic biopsychosocial interventions integrating medical, dietary, and psychological components for IBD management.

## 1. Introduction

Inflammatory Bowel Disease (IBD) is an umbrella term used to describe various chronic disorders that are characterized by excessive, relapsing, and prolonged intestinal inflammation. The two main IBD types concern Crohn’s disease (CD) and ulcerative colitis (UC) [1]. IBD affects over 3 million people in the United States and 2.5–3 million in Europe [2]. There is currently no known cure for IBD, meaning that the aim of the existing treatments is to induce remission and prevent recurrence [3]. IBD can negatively affect patients’ perceived physical and mental health status and quality of life. Given that patients often encounter several serious physical (e.g., pain, discomfort, surgeries), emotional (e.g., depression, anxiety, eating disorders), and social challenges (e.g., occupational issues, social isolation) [4].

Over the past years, there has been a growing interest in better understanding the underlying factors predicting IBD onset and prognosis. Past literature has examined a set of various genetic, epigenetic, and environmental factors predicting IBD, including but not limited to smoking, diet, drugs, geographical and social status, stress, microbial agents, sleep quality, and pharmacological influences [5,6]. Amongst those, diet and mental health constitute two significant modifiable factors that have been separately and thoroughly examined in relation to IBD.

In line with existing evidence, the most recent European Crohn’s and Colitis Organisation (ECCO) guidelines [7] also stress the important role of diet in the emergence of IBD, with Western-type diets having been related to an elevated risk of IBD onset. On a similar note, various dietary treatments have been developed over time for the management of UC and CD, with Exclusive Enteral Nutrition (EEN) currently being recognized by ECCO as the only established dietary treatment for CD [8]. More recently, precision nutritional therapy has emerged as an attractive therapeutic alternative in IBD management [9,10,11]. However, additional well-designed studies are needed to assess the effectiveness of dietary treatments and their subsequent use in clinical settings [9]. Likewise, specific recommendations for personalized dietary therapy options in IBD cannot yet be made, since the complex interplay between diet and gut inflammation should be further elucidated [10,11].

In relation to mental health, anxiety and depression are commonly observed among individuals living with IBD [12], with up to a third of patients affected by anxiety and a quarter by depressive symptoms [13]. Patients with IBD who are also living with anxiety or depression are at increased risk of hospitalization, emergency department visits, readmission, and used outpatient services more compared to their counterparts without mental health issues [2]. Recent evidence also emphasizes stress as a key environmental factor playing a crucial role in the pathogenesis and life-course of IBD. Consequently, effective management of stress and psychological symptoms may reduce inflammation and ameliorate disease-related adverse outcomes [14,15].

On the same note, the risk of developing eating disorders has been found to be high among IBD patients. As Cooney and colleagues showed (2024), young adults living with IBD are significantly more likely to develop eating disorders compared to their general population counterparts [16]. Eating disorders in IBD can have a detrimental impact upon the physical and mental health of people living with IBD, including malnutrition exacerbation, IBD management complications, increased risk for disease relapse, reduced quality of life, increased psychological distress, and heightened morbidity and mortality risk [7,17,18]. These overlapping psychological conditions have been shown to amplify disease burden and impair overall health in other chronic illnesses [19,20,21,22]; while this may also be relevant to IBD, the combined impact of anxiety, depression, and disordered eating has not yet been fully investigated in this population.

Although diet and mental health—particularly anxiety and depression—have been previously examined as independent factors in relation to IBD, their potential interplay has received less attention. The interplay between diet, mental health (particularly anxiety and depression), and IBD can be conceptualized as a complex and potentially bidirectional system, mediated in part through the gut–brain axis [23,24]. This axis describes the communication between the central nervous system and the enteric nervous system, linking emotional and cognitive processes with gastrointestinal function through neural, endocrine, and immune pathways [25,26]. Psychological factors such as anxiety, depression, and stress may influence gastrointestinal function by modulating immune responses and inflammatory processes. Conversely, intestinal inflammation in IBD may also affect mental health through neuroimmune pathways, including cytokine signaling. Diet represents an important, modifiable factor within this system, as it can influence gut microbiota composition and inflammatory status. In this context, dietary behaviors—including potentially disordered eating patterns—may interact with both disease activity and mental health outcomes [27,28,29]. At the same time, psychological distress may shape dietary choices and eating behaviors, suggesting the possibility of bidirectional relationships [30,31]. However, while these mechanisms are biologically plausible, the nature and extent of the interplay among diet, anxiety, depression, and IBD are only partially understood, highlighting the need for further research in this area.

Moreover, qualitative research shedding light on the subjective experience with the disease, particularly with regard to dietary behaviors and mental health, is scarce. The number of quantitative studies examining mental health outcomes at a larger scale in people living with IBD in Southern Europe and particularly in Greece is also limited. By combining large-scale quantitative data with patients’ perceived experiences of diet, mental health, and coping strategies in IBD, a better understanding can be achieved, ultimately informing interventions that reduce the disease’s impact on the lives of people living with IBD [32,33].

### Research Aim

Given the limited research examining the interplay between diet and mental health in people living with IBD from a holistic perspective, the present exploratory study adopts a mixed-methods design that integrates both quantitative and qualitative components. By combining standardized measures with in-depth insights into lived experiences, this study provides a more comprehensive understanding of diet and mental health within the Greek context. The quantitative component aims to (a) identify depression, anxiety, and disordered eating patterns in IBD patients in Greece, and (b) examine the interrelationships between disordered eating, anxiety, and depression. The qualitative component seeks to explore, in depth, patients’ perspectives on how diet and mental health interact in their lived experiences with IBD.

## 2. Materials and Methods

### 2.1. Study Design

An exploratory mixed-methods, parallel research design was employed, meaning that the qualitative and quantitative studies were concurrently conducted. The integration of quantitative and qualitative findings was primarily conducted at the level of interpretation, rather than through triangulation procedures or integration matrices at the analysis level. Findings of each study are separately presented but discussed together to shed light on the overall diet, mental health, and IBD experience of the participants.

Regarding the quantitative study component, a questionnaire survey was administered online to 283 IBD patients between July 2025 and February 2026. The online survey aimed to collect demographical information such as sex and diagnosis, alongside mental health outcomes, namely depression, anxiety and disordered eating, in IBD patients in Greece. Concerning the qualitative research component, 14 individual online interviews were conducted in the same time period to holistically and explore in depth the perceptions and views of IBD patients about the role of diet and mental health in their lived experiences with IBD.

### 2.2. Inclusion/Exclusion Criteria

Participants included in the quantitative and qualitative studies met the same basic inclusion criteria (age ≥ 18 years, diagnosis of IBD, ability to communicate in the Greek language and residency in Greece). No additional inclusion criteria were set.

### 2.3. Procedure and Ethical Approval

The current study gained ethical approval from the Ethics Committee of the Department of Nutrition and Dietetics, University of Thessaly. This study was also conducted in line with the code of conduct, legal regulations, and ethical guidelines defined by the University Ethics Committee (approval no: 67/10.12.2024). No adverse events were recorded during or after the completion of the online questionnaire survey or the individual interviews, meaning that both studies were completed as planned.

IBD patients were invited to participate in the online survey via social media pages and organizations that offer support to patients with IBD. The participants were provided with detailed information about the study objectives and procedures before providing informed consent. The online questionnaire survey included closed-ended questions about demographic data and standardized questionnaires to assess levels of anxiety, depression, and eating attitudes. It should be noted that data collection was anonymous, and no identifying information was collected, ensuring that responses could not be tracked back to individual participants.

Participants of the qualitative study were invited through two different routes. First, similar to quantitative study, interested individuals were invited to participate in both studies via social media pages and organizations that offer support to patients with IBD. Those interested contacted the research team directly. Second, IBD patients who had expressed interest in learning more about the interview study while completing the online questionnaire survey were given the option to declare their contact details within the online survey. The research team contacted these individuals and informed them about the scope of the qualitative study.

Next, in both recruitment pathways, the study information sheet explaining the scope of the study, participants risks and benefits, voluntary participation and confidentiality issues was circulated via email to those interested in participating in the interviews.

In the qualitative study, an individual online interview was scheduled and conducted on a day convenient for the participant and the researcher(s). Prior to starting the interviews, participants were required to sign off a consent form electronically. All interviews were conducted online. On the interview day, researchers, AV and DS, explained in detail the scope of the study and allowed time for participants to ask any questions. Interviews lasted about one hour. All interviewees had the opportunity to pause for questions or exit the interview earlier in case they experienced any type of psychological discomfort. It should be mentioned that all interviews were completed as planned, without any opt-out requests.

All data and information from both studies were in electronic form and remained confidential. Specifically, for the purposes of the quantitative study, no personal information (e.g., name) was collected, except for the email addresses of those participants who wished to take part in the qualitative study. The data of the participants were stored on computers of the Laboratory of Nutrition and Clinical Dietetics, after first being coded with numbers, in order to prevent personal data from being identified. The interview recordings were stored securely in an encrypted drive accessible only to the researchers of the studies. Interview data were transcribed verbatim prior to analysis. Data collected were then anonymized and uploaded to N-Vivo (Version 14) for analysis.

### 2.4. Materials

The online questionnaire consisted of closed-ended questions organized into sections covering demographic data and assessment of mental health of patients with IBD using valid questionnaires. All survey items were mandatory; therefore, there were no missing responses for the included participants.

#### 2.4.1. Generalized Anxiety Disorder Scale-7 (GAD-7)

Anxiety was measured with GAD-7 [34], a 7-item self-report scale that assesses the frequency of anxiety symptoms (e.g., “Feeling nervous, anxious, or on edge”) over the past two weeks on a four-point Likert scale from 0 (“not at all”) to 3 (“nearly every day”). The total score range is 0–21. Clinical significance is indicated by standard cut-offs: 5–9 = mild anxiety, 10–14 = moderate anxiety, and ≥15 = severe anxiety. The Greek version of the GAD-7 was used, which has demonstrated good internal consistency (Cronbach’s α ≈ 0.91) and confirmed single-factor structure [35].

#### 2.4.2. Patient Health Questionnaire-9 (PHQ-9)

Presence and severity of depressive symptoms was measured with PHQ-9 [36]. The instrument includes nine questions on a four-point Likert scale from 0 (“not at all”) to 3 (“nearly every day”) based on *Diagnostic and Statistical Manual of Mental Disorders, 5th Edition* (DSM-V) criteria for major depressive disorder (e.g., “Little interest or pleasure in doing things”). The total score range is 27 and standard clinical cut-offs are: 5–9 = mild, 10–14 = moderate, 15–19 = moderately severe, ≥20 = severe depressive symptoms. The Greek adaptation has been shown to be reliable and valid, with good internal consistency (Cronbach’s α ≈ 0.75) and appropriate factor structure [37].

#### 2.4.3. Eating Attitudes Test-26 (EAT-26)

Eating attitudes were measured with EAT-26 [38]. It includes 26 questions on a four-point Likert scale and assesses disordered eating behaviors. Participants indicate the extent of their agreement or disagreement with each statement as follows: 3 = always, 2 = usually, 1 = often, 0 = sometimes, rarely, or never (e.g., “I engage in dieting behavior”). The EAT-26 consists of three subscales: (1) dieting, (2) bulimia and food preoccupation, and (3) oral control. The total score ranges from 0 to 78, with a total score ≥ 20 indicating an increased risk for disordered eating. The Greek-adapted EAT-26 demonstrated good internal consistency, confirming its reliability for assessing eating attitudes in a Greek population [39].

It should be noted that PHQ-9, GAD-7, and EAT-26 constitute validated screening tools commonly used in clinical and research settings to assess severity of symptoms and identify individuals at potential risk; however, they do not constitute diagnostic instruments and should be interpreted alongside clinical evaluation.

#### 2.4.4. Interviews

A semi-structured topic guide entailing eight open questions was used for all interviews (Table 1). Questions explored IBD patients’ thoughts, beliefs and perceptions regarding the role of dietary behavior and mental health in their lived experiences with IBD. Probing and follow-up questions were used where appropriate to encourage deeper reflection and to clarify emerging topics.

### 2.5. Analyses

#### 2.5.1. Statistical Analysis

Categorical demographic data are presented as relative (%) frequencies. Data regarding the mental health outcomes of IBD patients (GAD-7, PHQ-9, and EAT-26 scores) are presented as median and Interquartile Range (IQR). Differences in the prevalence of symptoms between diagnostic groups, as well as between gender, were assessed using the Pearson Chi-square test. To compare symptom severity scores between patients with UC and CD, the non-parametric Mann–Whitney U test was employed, as data distribution was non-normal. The internal consistency and reliability of the GAD-7, PHQ-9, and EAT-26 scales were evaluated using Cronbach’s alpha (α) coefficients. Associations among depressive symptoms, anxiety and disordered eating attitudes in both patient groups were assessed using Spearman’s rank correlation coefficient (ρ). Statistical analysis was performed with Minitab 16. The analysis was descriptive and exploratory and did not test directional or predictive models. Given the exploratory approach of the current study, power calculation was not conducted prior to data collection.

#### 2.5.2. Thematic Analysis

A four-step thematic analysis, as described and explained by Braun and Clarke (2013) [40], was conducted to analyze the interview data. Thematic analysis was conducted inductively at a semantic level, focusing on participants’ experiences without imposing a pre-existing theoretical framework. Braun and Clark’s (2013) [40] four stages of analysis include the initial data familiarization, during which transcripts were read over multiple times and initial thoughts were recorded. Followed by the generation of initial codes, during which all data considered relevant were systematically coded across all transcripts. Then the searching for themes during which overlapping codes were combined to form themes. Lastly, the review of themes to ensure their compatibility with the entire dataset and the research questions. Finally, all themes were compared against existing literature to ensure a meaningful interpretation of the data. Data saturation was considered achieved when no new themes emerged from the final interviews, as assessed independently by the two coders [40]. Also, two researchers (DS, AV) independently coded the data and reviewed the resulting themes, discussing any discrepancies until reaching consensus. Researchers have backgrounds in psychology and nutrition, respectively. Both researchers reflected on their disciplinary perspectives and prior experiences with IBD during the analysis to enhance transparency.

## 3. Results

### 3.1. Quantitative Results

A total of 283 individuals (*n* = 173 CD, *n* = 110 UC) participated in the online survey. In both conditions, most participants were female (CD: 67.05%, UC: 70.00%). The median age was 41.50 years (IQR: 31.00–51.25) for the CD group and 45.00 years (IQR: 36.00–53.00) for the UC group. Regarding time since diagnosis, the largest proportion in both groups reported disease duration ≥ 11 years (CD: 42.77%, UC: 41.82%), while approximately one-third of CD patients (30.06%) and one-fourth of UC patients (23.64%) had been diagnosed in the past 1–5 years. The majority of participants resided in urban areas (CD: 89.6%, UC: 83.64%) and were currently taking medication for IBD (CD: 90.17%, UC: 93.64%), while use of biologic agents was reported by 67.05% of CD patients and 54.54% of UC patients. Recent medication change was reported by 17.92% of CD patients and 21.82% of UC patients. History of surgery was more common in the CD group (29.48%) compared to the UC group (6.36%). Table 2 presents the descriptive characteristics of the questionnaire survey participants’ in greater detail.

In the present study, the GAD-7, PHQ-9, and EAT-26 demonstrated high internal consistency, with Cronbach’s alpha coefficients of 0.934, 0.893 and 0.917 respectively.

With regard to the mental health outcomes, as shown in Table 3, moderate to severe depression was recorded in 42.20% of the CD group and 54.54% of the UC group. Moderate to severe anxiety levels, as depicted in GAD-7, were reported by 36.99% of CD patients and 54.55% of UC patients. respectively. Finally, according to EAT-26, a high risk of eating disorder was identified in 27.17% of CD patients and 27.27% of UC patients.

Detailed comparative analysis of clinical symptoms is presented in Table 4. Differences were observed in the prevalence of mental health symptoms between the two diagnostic groups. According to the Chi-square test, a higher proportion of patients with UC exhibited moderate to severe anxiety (54.55% vs. 36.99%, *p* = 0.004) and moderate to severe depression (54.54% vs. 42.20%, *p* = 0.042) compared to those with CD. In contrast, disordered eating attitudes were low across the entire sample, with no significant difference observed between the two diagnostic groups (*p* = 0.985).

Regarding the symptom severity, as shown in Table 5, the Mann–Whitney U test indicated that patients with UC reported significantly higher anxiety scores (Median = 10.5) than patients with CD (Median = 7.0, U = 7920, *p* = 0.017). While the median depression score was also higher in the UC group (11.0 vs. 8.0), this difference was not statistically significant when comparing overall ranks (U = 8353, *p* = 0.083). However, regarding disordered eating attitudes, the Mann–Whitney U test showed no significant difference between diagnostic groups (U = 9505, *p* = 0.648).

To address the potential impact of confounding variables, the two diagnostic groups were comparable in terms of age (*p* = 0.059) and gender (*p* = 0.868). However, a statistically significant difference was observed in residence (*p* = 0.036), with patients residing in rural areas being more likely to have UC.

Because of the female predominance in our sample and the possible influence of pharmacological treatment, we conducted a subgroup analysis regarding anxiety, depression and disordered eating attitudes. Chi-square tests revealed no statistically significant differences between male and female participants in the prevalence of moderate to severe anxiety (*p* = 0.872) or depression (*p* = 0.707). Although a higher descriptive trend was noted in females regarding high-risk eating attitudes (31.1% vs. 19.5% in males), this difference did not reach statistical significance (*p* = 0.075). Similarly, no significant differences were observed across residential subgroups in the prevalence of moderate to severe anxiety (*p* = 0.338), depression (*p* = 0.095) or high-risk eating attitudes (*p* = 0.884). Pharmacological treatment did not significantly differentiate the prevalence of anxiety (*p* = 0.632), depression (*p* = 0.682) or disordered eating (*p* = 0.500). Finally, there were no significant differences regarding the prevalence of anxiety (*p* = 0.559), depression (*p* = 0.659) or disordered eating (*p* = 0.844) according to self-reported disease activity status (relapse vs. remission).

Spearman’s correlation analysis revealed statistically significant positive correlations among depressive symptoms, anxiety and eating attitudes in both patient groups (see Table 6 below). In more detail for the CD group, a mild to moderate positive correlation was observed between PHQ-9 and EAT-26 (ρ = 0.317, *p* < 0.001). GAD-7 was also positively correlated with EAT-26 (ρ = 0.241, *p* = 0.001), although the correlation was weaker. In addition, a strong positive correlation was recorded between PHQ-9 and GAD-7 (ρ = 0.719, *p* < 0.001). Similarly, in the UC group, PHQ-9 and EAT-26 were mildly positively correlated (ρ = 0.278, *p* = 0.003), while a similar mild positive correlation was observed between GAD-7 and EAT-26 (ρ = 0.256, *p* = 0.007). The correlation between PHQ-9 and GAD-7 was stronger and statistically significant too (ρ = 0.662, *p* < 0.001).

### 3.2. Qualitative Results

Fourteen IBD patients participated in the qualitative study. It should be noted that qualitative research tends to use smaller samples and there are no specific rules regarding the sample size [40]. As such the sample was sufficient to support meaningful thematic development within the scope of this exploratory study. All participants were living with IBD, and specifically with CD (*n* = 9) or UC (*n* = 5). Their median age was 44 years. Men and women were equally represented. Most interviewees lived in urban areas. All participants were educated to at least the secondary education level, and most were employed. The majority received their diagnosis at least 11 years ago and were in remission. Table 7 presents the descriptive characteristics of the study participants in greater detail.

**Figure 1:**
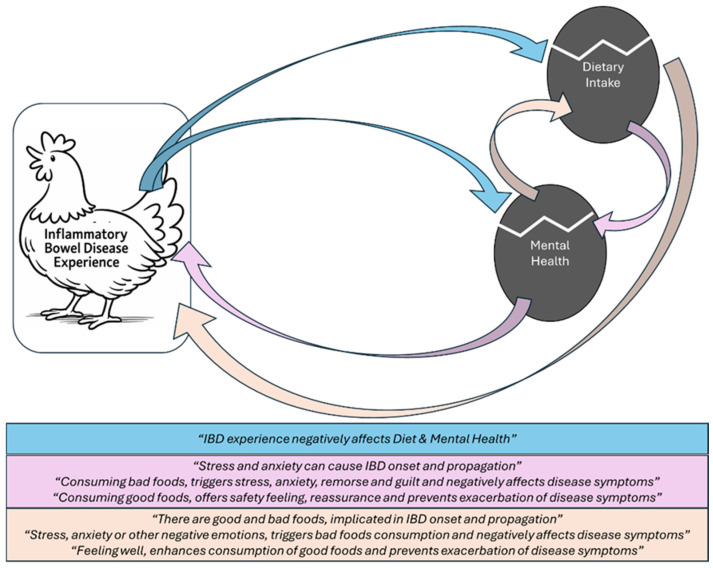
Graphic representation of the thematic analysis results.

Three key themes emerged from the analysis namely theme 1: ‘Life in two phases: IBD impact on health’, theme 2: ‘Mental health and diet interplay—their perceived impact on IBD’ and theme 3: ‘Coping strategies for managing IBD’. Overall, no differences were observed in the results as to key demographic characteristics including the disease status, meaning that UC and CD patients shared similar beliefs under each theme. As described below in most themes, overall disease experience was strongly shaped by the disease activity status which affected both the physical and mental health of both patients with UC and CD. During relapses, some participants experienced symptoms such as pain, fatigue, and loose stools, and restricted diet, physical activity and social functioning; some also experienced increased anxiety and depression. On the contrary, periods of remission were accompanied by improved mood and increased reintegration into daily life and social activities. Nevertheless, the experience of repeated flare-ups maintained a persistent sense of vigilance. According to participants, mental health was strongly associated with diet; stress could trigger or exacerbate symptoms, while dietary choices influenced the psychological well-being and physical functioning of the IBD patients (Figure 1). As most participants explained, efforts to maintain a sense of control over their lives and, thus, better manage their disease, involved a combination of internal coping strategies, such as dietary adjustments, and external sources of formal and informal support, including family, friends and healthcare professionals and patients’ forums. Each theme is separately and in-depth described below.

#### 3.2.1. Theme 1: Life in Two Phases: IBD Impact on Health

Within this theme, all participants reported that the experience and phase of the disease affected multiple aspects of their lives, shaping their physical and mental health as well as their social functioning. The phase of the disease (i.e., remission or relapse) was often mentioned as an important determining factor for their emotional state, dietary behaviors, and social identity. Many participants emphasized that the period of relapse was often accompanied by multiple restrictions, heightened alertness and anxiety, as well as social withdrawal. In contrast, periods of remission were frequently experienced as phases of relief and as a temporary restoration of “normality”.

#### 3.2.2. Subtheme 1.1: Physical Health and Health Behaviors

Changes in the body and in one’s relationship with food represented a central aspect of the IBD experience for almost all participants. Many participants described the experience of physical pain during a flare-up as a central issue. The pain was described as intense, persistent and unpredictable, associated with diarrhea, bleeding and gas, which significantly disrupted their daily lives. Beyond physical suffering, pain was reported as an experience of exposure and vulnerability, especially in social contexts. As one participant characteristically stated:

*“It’s not just the pain, it’s that you don’t know when it’s going to hit you—and that keeps you constantly on edge.”* (PART_IBD8, Male, Crohn’s Disease)

Almost all participants highlighted the pivotal role of diet in both the intensity and management of their symptoms, noting that their eating habits change depending on the phase of the disease. More specifically, as many explained, during the relapse phase, diet becomes more restrictive, with an emphasis on tolerable foods that do not aggravate symptoms. For example, during relapses, food choices are limited to minimal and repetitive options, such as rice or boiled chicken. Eating is experienced as a means of survival rather than as a source of pleasure.

*“I was not well, I could not eat much, I ate less … then I developed a fear of food, because when I was in a flare, no matter what I ate, I had 15–20 bowel movements a day.”* (PART_IBD11, Female, Ulcerative Colitis)

In contrast, remission was associated with greater dietary flexibility and gradual food reintroduction. Several participants noted that during remission, symptoms subside and patients do not impose major dietary restrictions.

*“While I am in remission I eat everything, I enjoy life to the fullest and food is an important part of my socialization.”* (PART_IBD14, Female, Crohn’s Disease)

For some interviewees, though, this dietary freedom was accompanied by a strong desire for food, which in certain cases led to binge or overeating behaviors, as a reactive response to previous restrictions. For other interviewees, the experience of repeated flare-ups left a heightened sense of fear, threat, and constant alertness; these interviewees mentioned that they continued to avoid certain foods or social situations out of fear of relapsing, suggesting a persistent internalized sense of physical vulnerability.

A similar pattern was observed with physical activity. Many patients described discontinuing exercise during flare-up phases, due to fatigue and exhaustion. During remission, though, they returned to their usual physical activities such as running and these were experienced as a sign of regained health and strength.

#### 3.2.3. Subtheme 1.2: Mental Health, Social Life and Relationships with Significant Others During Relapses

For many interviewees, mental health mirrored their disease phase. Relapses were associated with increased anxiety, low mood and social isolation whereas remissions were characterized by ‘small breaths of relief’, rejuvenation and improved emotional well-being.

*“When in a flare-up, I felt withdrawn, I would say, I couldn’t communicate with those close to me, I didn’t have the mood. I was quite isolated at that time.”* (PART_IBD4, Female, Crohn’s Disease)

Moreover, during relapses, the participation of people with IBD in social and professional activities was negatively affected. As one participant said:

*“As I said, because of the physical symptoms and the pain I had when I relapsed, I was excluded from any social activity, not related to my school or work, I didn’t go out with friends because even if I did I couldn’t communicate with my friends, I was constantly lost. I couldn’t communicate.”* (PART_IBD4, Female, Crohn’s Disease)

According to participants, flare-ups frequently resulted in work absenteeism and social withdrawal, due to the fear of symptoms appearing in public and the lack of available and accessible facilities (e.g., clean toilets). As many participants explained, this need for safety and control often led them to seek their “shelter of home”, significantly limiting their social life. Moreover, some interviewees often felt ‘misunderstood or not understood’ by friends and relatives, which contributed to feelings of loneliness.

*“However, I think that the pain, after all, I woke up alone, I slept alone, so I think that unfortunately, like all illnesses, the patient goes through it alone. I mean, I experienced it a little bit alone and because I don’t want to burden others, maybe I even distanced myself from them so as not to burden them. In other words, I didn’t want to talk to my friends. I talked to very specific people. I didn’t want to upset them or burden them. Therefore, I explained to them that I wasn’t in a good mood, a little more distant.”* (PART_IBD11, Female, Ulcerative Colitis)

Some interviewees, particularly the younger ones, when in relapse discussed the impact and challenge of explaining symptoms to their partners. A couple of participants even ended romantic relationships due to concerns about potential “embarrassing” situations or due to the difficulty of their partners in understanding the nature of their symptoms.

*“I didn’t have any sexual contact because I was worried that something like what you said would happen. So I was very limited in the sexual aspect as well.”* (PART_IBD4, Female, Crohn’s Disease)

#### 3.2.4. Theme 2: Impact of Mental Health and Diet on Disease

According to all participants, mental health played a crucial role in the onset and the deterioration of their symptoms. Many also discussed how their mental health may affect their dietary habits and food choices, which in turn may influence their perceived symptoms; fluctuations in symptom severity then fed back into their mood and eating behaviors, creating a vicious cycle between mental health, dietary patterns, and perceived disease experience.

#### 3.2.5. Subtheme 2.1: Stress as a Trigger for the Disease Diagnosis

Many participants mentioned that the onset of the disease was influenced by intense psychological pressure and stress they had previously experienced. As they pointed out, the first symptoms emerged and the diagnosis was made following a period of intense stress, low mood, and extreme fatigue. As one participant said:

*“When the diagnosis was made and a psychologist came to talk to me at my first hospitalization and we discussed some things, I basically understood that the intense stress I had been going through in the previous months might have been the cause that triggered this disease and started to bother me.”* (PART_IBD7, Male, Crohn’s Disease)

#### 3.2.6. Subtheme 2.2: The Role of Stress in Symptoms

Many interviewees said that psychological pressure and stress were determining factors for their symptoms. Psychological tension often manifested physically as increased pain, cramps and discomfort in the intestine, affecting interviewees’ daily functioning and quality of life. Periods of heightened emotional pressure precipitated the onset and intensity of symptoms.

*“I strongly believe that stress plays a major role in my symptoms. As I said, I felt that if I were to be stressed for some reason, if something stressful was coming, I would start experiencing pain and bloating.”* (PART_IBD4, Female, Crohn’s Disease)

#### 3.2.7. Subtheme 2.3: Impact of Different Food Categories on the Disease

Participants expressed mixed thoughts about the impact of various food categories on their symptoms. Although many acknowledged that certain foods could exacerbate or even trigger symptom flare-up, this relationship was not perceived as unilateral but instead as a result of ongoing testing, observation, and personal experience.

Specifically, no differences were observed between patients with UC and CD in terms of perceptions of food. Instead, differences emerged based on disease activity: all participants during flare-ups restricted the consumption of certain foods, while during remission, they were more flexible and incorporated a greater variety of foods into their diet.

Foods that were generally considered safe during outbreaks by most participants included rice, potatoes, and boiled chicken. Overall, some boiled vegetables and fruits had a mixed effect for some, while foods such as legumes, fried foods, fatty foods, and lactose-containing dairy products were associated with negative symptoms for others.

*“In my early years with the disease I was constantly in flare-ups and there are specific foods that you can eat when you have a flare-up, namely pasta, rice, boiled chicken, for example, boiled potatoes, meaning you can’t eat greens, vegetables or many fruits, because they affect your intestines when you have inflammation, they essentially make you worse, I can’t go eat lettuce, let’s say, it will shake me, that is, where I can go, with the exacerbation, 20 bowel movements a day, if I eat lettuce I will go 25–30.”* (PART_IBD7, Male, Crohn’s Disease)

During remission, consumption expanded to include fish, eggs, and more fruits and vegetables, while the negative effects of processed and high fat foods, fried foods, and lactose-containing dairy products remained for most interviewees. Several participants reported a positive effect from foods such as fruits, vegetables, probiotics (kefir, lactose-free yogurt) and supplements such as curcumin, especially during periods of remission.

*“… Since I took curcumin pills, because I was also a little gassy, bloating, when I started it after a month I was quite well, so I have continued it until now.”* (PART_IBD3, Male, Ulcerative Colitis)

Some participants also reported that proper food preparation (e.g., soaking legumes), when in remission, could reduce intestinal discomfort.

*“Because, let’s say, legumes are disastrous. However, under certain conditions, lentils, for example, since they have been cooked in a special way and so on, have fewer side effects, at some point I will eat them, although I shouldn’t. Why? Because it’s what you’re missing, and you want it.”* (PART_IBD2, Male, Ulcerative Colitis)

Table 8 summarizes the food categories and participants’ perception of feedback as positive, negative, or neutral, with differentiation according to disease activity (flare-ups vs. remission). The results show that, while the general trend is influenced by disease status, some foods are widely considered safe or restrictive regardless of UC or CD.

#### 3.2.8. Subtheme 2.4: The Stress-Nutrition-Disease Relationship

Many participants reported that their mental health impacted their eating behavior. During periods of heightened stress patients would alter their dietary behavior to manage their stress. On the contrary, a positive mood tended to promote healthier nutritional behaviors that some participants associated with symptom remission.

*“I understand, let’s say, that when I have a good day and I don’t have any symptoms, I’m happier, so I’ll eat better, or if I’ve eaten well one day, if I’ve eaten according to the program one day and I don’t have any physical discomfort, I’ll be happier with myself. And I’ll feel that I’m actually more active, more functional, I’ll go out more easily, I’ll be more social, I’ve definitely noticed that.”* (PART_IBD4, Female, Crohn’s Disease)

On the other hand, the consumption of certain foods seemed to affect patients’ emotional wellbeing by intensifying or soothing negative emotions. For instance, following a diet rich in “safe” foods—as these were described above—would foster psychological stability and would reduce disease symptoms.

*“Look, when you don’t feel well, it can often mean that you’re psychologically bad, so by extension you don’t do some things like cook properly, eat the right amounts and times, so you may resort to something that’s outside, which isn’t so good for our part, because okay, there are certainly shops that I don’t mind at all and I’ve tried them, but I’ve happened to be in some areas that I don’t know, so I’m forced to use some other shops that then create a problem for me.”* (PART_IBD6, Female, Ulcerative Colitis)

Thus, an interdependent cycle was described as participants reported, where disease symptoms were mutually and concurrently shaped by the interaction of mental health and dietary behavior.

#### 3.2.9. Theme 3: Perceived Coping Strategies with the Disease

Within this theme, all participants described internal and external coping strategies for managing their disease. Internal coping strategies referred to individual mechanisms, practices and behaviors employed to manage the disease. External coping strategies, on the other hand, referred to the support received from the external environment (e.g., from friends, relatives, or institutions) that, in turn, facilitated effective disease management.

#### 3.2.10. Subtheme 3.1: Internal Coping Strategies

Dietary modifications were described by almost all interviewees as crucial strategies for disease management. For instance, adopting a balanced and personalized diet could enhance symptom control. Similarly, avoidance of certain foods as described above could preserve remission.

*“Now that I am in remission, I have also entered a program. I avoid all red foods for both nutrition and the gut, so I am very well. I do not spoil it, since I have found that this works, I will continue with it.”* (PART_IBD3, Male, Ulcerative Colitis)

Many participants discussed how fasting or food avoidance in certain social situations such as traveling constitutes an efficient strategy for retaining remission. According to those participants, food avoidance and fasting minimized the likelihood of severe gastrointestinal symptoms in situations of limited access to appropriate food or medical care.

*“When I had to go to work, because my job is far from home, I have to drive there, it’s about 1 h, so I wouldn’t eat before. Many times when I was at work and I knew that I had an intense daily routine, I still wouldn’t eat at work at all. When I was traveling to come to Athens, … , a three-hour drive before, I didn’t eat, not even during the trip. When I was going out with friends, okay, because I knew that I could escape if I had an intense need to defecate. Generally, when I felt like I was going to leave and I wouldn’t have a toilet next to me, I didn’t do it. I didn’t eat at all. I think those were the situations. And when I had intense abdominal pain, I also didn’t eat.”* (PART_IBD4, Female, Crohn’s Disease)

Alongside dietary modification, regular physical activity was identified, by most participants, as an important internal coping strategy. According to some interviewees, physical exercise reduces stress and consequently symptoms intensity, by providing a form of psychological relief. Physical activity also increased the sense of control over the body, contributing to improved symptoms and pain management.

*“But I would start again very immediately (exercise), if the gym had not closed, because it helps me. I get away from the same old things, my psychology improves, I forget that I have an issue, it seems to me that I am the same as the rest. Mainly, I feel better about my body. That is, I feel that the bloating that we have from food is not so noticeable, because when the body changes, I think you get even more depressed when you are used to something else and you are constantly swollen and in pain. And the pain also decreases, basically when I do exercise, it seems to me that the pain decreases.”* (PART_IBD6, Female, Ulcerative Colitis)

As many participants explained, accepting the chronic nature of the disease is a crucial step in achieving remission and improving quality of life. Psychotherapy emerged as a central mechanism of acceptance and adaptation as it helped patients to conceptualize themselves beyond the confines of their illness, regain self-esteem, boost sense of control over life, manage stress and address psychosomatic symptoms.

*“I had some image issues because I have scars on my torso and so on, and a little with the psychologist, a little with the support of my family, I overcame them. In general, I think it (psychotherapy) helps me to see myself as a person, regardless of the chronicity of the disease.”* (PART_IBD5, Female, Crohn’s Disease)

#### 3.2.11. Subtheme 3.2: External Coping Strategies

According to most interviewees, a supportive environment acted as a catalyst in managing the disease. Patient communities, such as online forums, could provide emotional support and alleviate feelings of isolation.

*“Yes (I find support on a patient forum) very much. It’s just that I calm down, I stop being tense. There are days when I get off work, and I’ve realized that my stomach is tense as if I’ve been doing sit-ups for I don’t know how long, which is definitely not good for Ulcerative Colitis.”* (PART_IBD3, Male, Ulcerative Colitis)

Similarly, family and friends provided support and a sense of being understood, which improved resilience and contributed to symptom stability. In particular, family support provided a sense of safety and could facilitate daily adaptation to the demands of IBD.

*“… just like I had said, as I had experienced because I was in chaos, I couldn’t cope socially, but my family definitely supported me a lot and it was very important to have them by my side.”* (PART_IBD4, Female, Crohn’s Disease)

Some participants highlighted the role of healthcare professionals as fundamental in disease management. Collaboration with doctors, dietitians, fitness trainers and psychologists was perceived as central to a holistic care approach that should be characterized by mutual trust and frequent communication.

*“For some reason, I was taking more vitamins, whatever the doctors told me. I had become very attached to my doctor, and I was very insecure at that time, so I was trying to find a way to bridge the communication with the doctor. I also have my psychologist and I had intensified the sessions a little. Because under other circumstances it would have been 2 weeks, I did it weekly. In an attempt to understand what was happening to me.”* (PART_IBD4, Female, Crohn’s Disease)

On the other hand, a few participants stated that they did not receive the level of support they needed from healthcare professionals; these participants explained that they were offered inadequate disease-related guidance and limited time for personal interaction due to the increased workload of the healthcare professionals.

*“In summary, I think that, because my disease is chronic, as a joking professional patient, who has now learned the communication codes, the systems, it was not easy, you know, the deprivation of dignity when you enter a public hospital is inevitable, it is not an end in itself for others, but professionals cannot treat you well with such a workload.”* (PART_IBD9, Male, Crohn’s Disease)

## 4. Discussion

This is the first mixed-methods study conducted in Greek patients with IBD to explore the relationship between diet, mental health, and disease experience. Taken together, our findings offer a key novel contribution through their holistic and in-depth exploration of the complex interplay between mental health and diet across disease onset and progression.

Our quantitative findings indicate that a substantial proportion of individuals with IBD experience heightened anxiety and depression, consistent with previous literature reporting a high psychological burden in this population [2,41]. However, in contrast to prior research and our qualitative findings, which both suggest that psychological distress intensifies during periods of active disease, our quantitative results did not reveal significant differences between relapse and remission subgroups. This may indicate that, while disease activity shapes the experience and expression of distress, the overall psychological burden remains relatively persistent across disease phases. In line with this, qualitative accounts showed that relapse was associated with fatigue, dietary restriction, hypervigilance, and social isolation whereas remission with relative relief and re-engagement; however, when in remission most interviewees often reported a lingering sense of vulnerability and heightened awareness.

Notably, all three conditions, namely, depression anxiety and disordered eating, were positively interrelated in both the UC and CD patients, indicating that these conditions may co-occur in IBD patients. This pattern aligns with our qualitative results, according to which interviewees described an interdependent relationship between psychological distress, dietary behavior, and symptom experience. Specifically, emotional distress was perceived by almost all interviewees to influence eating behaviors and symptom perception, while dietary choices were simultaneously viewed as shaping both gastrointestinal symptoms and emotional well-being. Qualitative participants also described fluctuating cycles in which psychological stress, dietary restriction or modification, and symptom exacerbation reinforced one another, alongside the use of both internal and external coping strategies to regain a sense of control.

This integrated pattern is consistent with a broader “syndemic” perspective, whereby co-occurring mental health symptoms and disordered eating behaviors interact to amplify overall disease burden. For instance, in individuals living with obesity the coexistence of depressive symptoms, anxiety, and emotional overeating appears to exacerbate weight gain and metabolic dysfunction, resulting in a reinforcing cycle of psychological and physical health risks [42]. Similarly, in individuals living with type 2 diabetes, anxiety and depression are associated with reduced dietary adherence, which jointly increases the risk of overall health complications [43]. The aforementioned examples suggest that the combined effect of mental symptoms and disordered eating patterns may amplify the burden of disease, highlighting the need for comprehensive interventions that target both mental health and lifestyle behaviors in chronic conditions, and probably in IBD.

In our study, patients with UC exhibited significantly higher levels of psychological distress than patients with CD, including increased prevalence of anxiety and depression as well as elevated anxiety severity scores, suggesting that disease-related factors in UC may be associated with increased psychological vulnerability. These findings may suggest that the clinical manifestations or specific symptoms associated with UC in our sample could lead to greater psychological distress compared to CD. While the qualitative analysis did not reveal clear differences between UC and CD in perceived psychological burden, it still provided significant contextual insight into the mechanisms underlying the increased psychological burden observed quantitatively in UC patients. In particular, participants across both groups described how symptom unpredictability, disease relapses, and social restrictions contribute to augmented emotional distress. These experiences, though, might well be more impactful in conditions characterized by greater urgency and social interference, such as UC.

Despite the strong correlations between mental health and eating behaviors in our survey sample, the prevalence of high-risk eating attitudes remained comparable across all patients. This finding suggests that while anxiety and depression may be diagnosis-dependent, disordered eating attitudes appear to be a more generalized challenge for IBD patients, potentially linked to the universal dietary concerns inherent in IBD management. Likewise, qualitative findings showed how most interviewees adapted their diet based on strong personal beliefs about “safe” or “trigger” foods for IBD symptoms exacerbation, frequently in the absence of evidence-based dietary guidance from healthcare professionals. These distorted dietary perceptions were often embedded within broader coping processes, where restrictive eating patterns reflected a vicious cycle between symptom management, distress, and perceived control over IBD. These findings are consistent with previous research showing that such beliefs affect food-related quality of life [24]; they further highlight the need for psychoeducation for patients and targeted training for healthcare professionals, including dietitians and physicians, to address misconceptions about diet, support adaptive eating behaviors, and facilitate early identification of individuals at risk of maladaptive eating patterns in IBD [44,45].

Although our sample was characterized by a female predominance, subgroup analyses confirmed that this factor did not fundamentally bias our primary outcomes. The prevalence of anxiety, depression and disordered eating attitudes remained consistent across genders, suggesting that the reported psychological burden and eating behaviors are not artifacts of gender imbalance. This finding contrasts with previous studies reporting higher levels of anxiety, depression and disordered eating attitudes among female individuals living with IBD compared to their male counterparts [13,41,46,47]. One possible explanation is that the female predominance in the present sample may have attenuated detectable gender differences. Also, it is possible that gender-related differences in symptom reporting or health-seeking behavior may have contributed to this finding.

Similarly, although the UC and CD groups differed significantly in their residential distribution, residence was not found to be associated with psychological outcomes, indicating that the higher levels of anxiety observed in UC patients were independent of their living environment.

These quantitative findings are further supported by our qualitative analysis, which did not reveal differences in participants’ experiences across gender or residence, with perceived distress and coping strategies primarily driven by disease activity rather than sociodemographic factors.

Overall, our mixed-methods findings are consistent with a growing body of evidence emphasizing the bidirectional communication between the gut and central nervous system. The gut–brain axis framework highlights how stress, diet and gut inflammation are interconnected via neural, hormonal, metabolic, immunological and microbial signals [30]. Various stressors are known to alter or dysregulate the “gut–brain” axis and potentially worsen the course of IBD [48]. Activation of the hypothalamic–pituitary–adrenal (HPA) axis among other pathways is linked with these alternations [30,49].

Building on this mechanistic understanding, our findings support the need for a holistic, biopsychosocial approach regarding the effective management of IBD. Instead of solely focusing on pharmacological therapies, our results underscore the importance of taking into consideration the dynamic interplay between diet, well-being, and disease activity. This perspective aligns with the emerging evidence advocating for precision medicine, and calls for a more tailored and individualized approach which incorporates non-pharmacological factors—such as stress, lifestyle, and diet—into comprehensive IBD management and care [50,51]. In this context, the integration of non-medical interventions (e.g., psychological or behavioral interventions) can be highly beneficial for the effective management of IBD [15,50,52,53,54,55]. For instance, cognitive behavioral therapy (CBT) has been linked to improved mental health outcomes in IBD patients [15,53,54,55], whereas psychogastroenterology may contribute to the enhanced management of gastrointestinal symptoms [55,56].

Taken together, our findings provide contextually grounded evidence from a Greek population and could further inform the development of holistic healthcare models and interventions that are culturally sensitive and relevant to patients’ lived experiences and include both nutritional and mental health elements (e.g., nutritional counseling and psychoeducation). Holistic models of medical and psychosocial support may support the provision of personalized IBD care and increase medical adherence and patient satisfaction [14].

This study has several strengths but also some key limitations. The greatest advantage of this study concerns its mixed-methods research design. By combining complementary quantitative and qualitative methods, the strengths of each are maintained, while at the same time their relative limitations are weakened. As a result, a more holistic and comprehensive understanding of a certain phenomenon is attained, in a way that the implementation of only either a qualitative or a quantitative approach would not allow [57,58]. Moreover, another advantage concerns the sufficient total sample size participating in the survey and the interviews. The semi-structured interview approach allowed for flexibility and IBD patients’ voices to be heard whereas the use of validated and widely recognized psychometric survey tools ensured reliability and comparability with the international literature.

On the other hand, the cross-sectional design of the present study limits causal inferences. The lack of methodological integration between the qualitative and quantitative components at the analysis level constitutes another limitation of the study. The convenience sampling strategy and the exclusive recruitment of study participants through patient associations and social media platforms introduce the potential for digital self-selection bias, as individuals who are more active in online communities or seeking emotional support may have been more likely to participate. Consequently, the sample may be skewed toward participants with an elevated psychological burden, potentially distorting the observed relationships between diet, mental health, and IBD. Additionally, this recruitment strategy may have led to the overrepresentation of more engaged participants, as well as those experiencing greater psychological distress. As a result, the prevalence of depression, anxiety, and disordered eating may be overestimated, and the findings should therefore be interpreted with caution.

The lived experiences of IBD patients living in small or remote places (e.g., small villages) have not been sufficiently captured in the qualitative component of the study; these results, thus, may not be applicable to different populations across Greece. Another limitation innate to qualitative methods concerns researchers’ susceptibility to interpretive bias. To mitigate this limitation during the analysis stage, two researchers (DS, AV) independently coded the data and reviewed the resulting themes, discussing any discrepancies until reaching consensus.

With regard to the quantitative component, online data collection broadened the geographical coverage and facilitated the participation of patients from all over Greece; however, the predominance of urban participants may introduce urban residence bias, potentially limiting the generalizability of the findings, particularly to rural populations. Another limitation is the lack of objective measures of inflammation, such as fecal calprotectin or C-reactive protein, to validate the self-reported disease activity and symptoms. As a result, the associations among mental health, dietary behaviors, and disease activity are based on patient perceptions and may not fully reflect biological inflammation. No formal sample size calculation was conducted; therefore, it cannot be definitively established whether the study was optimally powered to detect all potential associations. While the relatively large sample provides a reasonable basis for exploratory analyses, the possibility of type II error and less stable effect estimates cannot be fully excluded. Furthermore, the absence of multivariate analyses controlling for confounders potentially limits the identification of independent associations, as the observed associations may be influenced by unmeasured or uncontrolled variables. Although some descriptive differences between UC and CD groups were observed, the study was not designed to explain these differences. In this context, future quantitative research may also benefit from multivariate regression models to further adjust for potential confounders and explore the independent predictors of psychological burden in IBD.

## 5. Conclusions

This mixed-methods study provides novel insights into the complex interplay between the diet, mental health, and disease experience of individuals living with IBD. Psychological distress may be high among IBD patients and closely interlinked to dietary perceptions and behaviors which are also being shaped by the unique lived experiences and coping strategies of IBD patients. Our findings underscore the importance of shifting our focus from purely pharmacological to holistic and biopsychological approaches that integrate dietary and psychological factors in IBD management and care. Future longitudinal studies are necessary to further investigate causal mechanisms and better inform the IBD clinical care.

## Figures and Tables

**Table 1:** Topic guide for conducting semi-structured interviews with adult IBD patients.

Questions of the Semi-Structured Topic Guide
Question 1. In what way has your diet changed after your diagnosis, if it has changed at all? (For example, changes in quantity, types of food, cooking methods, changes in consumption of food categories/groups, more/less takeaway food.)
Question 2. What role does your diet play in your illness? (For example, symptoms, flare-ups/remission.)
Question 3. In what way does your diet change depending on whether you are in a flare-up or in remission? (For example, foods you prefer or avoid, or changes in eating habits.)
Question 4. What helps you manage your illness effectively? (Which support sources do you rely on? For example, support from friends, family, or formal sources such as psychotherapy or patient forums.)
Question 5. What can be helping your mental health? (This may include formal sources of support—e.g., healthcare professionals—and informal sources such as friends, activities, or social media.)
Question 6. What do you feel may burden your mental health? (For example, changes in social life, changes in your body, changes in your relationships.)
Question 7. How do you think your dietary habits are related to your mental health within the context of your illness? (For example, a cause–effect relationship—“when I don’t feel well, I eat differently” or “I eat differently in order to feel better.”)
Question 8. How do you think the relationship between mental health and diet affects your overall IBD experience, if it affects it in any way? (For example, do they contribute equally or interact with each other?)

**Table 2:** Demographic characteristics of IBD patients, who participated in the questionnaire survey.

	CD (*n* = 173)	UC (*n* = 110)
Gender		
Female	67.05%	70.00%
Male	31.79%	29.09%
Prefer not to say	1.16%	0.91%
Age (median, IQR)	41.50 (31.00, 51.25)	45.00 (36.00, 53.00)
Time since diagnosis		
0–6 months	3.47%	4.55%
7–12 months	4.05%	3.64%
1–5 years	30.06%	23.64%
6–10 years	19.65%	26.36%
11 years or more	42.77%	41.82%
Area of Residence		
Rural area	10.4%	15.45%
Urban area	89.6%	83.64%
Prefer not to say	0.00%	0.91%
Current IBD medical treatment		
Yes	90.17%	93.64%
No	9.83%	6.36%
Current use of biologics	67.05%	54.54%
Recent change in medical treatment		
Yes	17.92%	21.82%
No	82.08%	78.18%
History of surgery		
Yes	29.48%	6.36%
No	70.52%	93.64%

**Table 3:** Severity distribution of depressive symptoms (PHQ-9), anxiety symptoms (GAD-7), and eating disorder risk (EAT-26) in CD and UC groups (within-group column percentages).

**PHQ-9**	**CD (n = 173)**	**UC (n = 110)**
Minimal depression	30.64%	20.00%
Mild depression	27.17%	25.45%
Moderate depression	17.92%	25.45%
Moderately severe depression	12.72%	19.09%
Severe depression	11.56%	10.00%
**GAD-7**	**CD (*n* = 173)**	**UC (*n* = 110)**
Minimal anxiety	30.06%	20.00%
Mild anxiety	32.95%	25.45%
Moderate anxiety	16.18%	30.91%
Severe anxiety	20.81%	23.64%
**ΕAΤ-26**	**CD (*n* = 173)**	**UC (*n* = 110)**
High Risk	27.17%	27.27%
Low Risk	72.83%	72.73%

**Table 4:** Prevalence of clinical symptoms by diagnosis (N = 283).

Variable	CD (*n* = 173)	UC (*n* = 110)	χ^2^	*p*
Moderate to severe anxiety (GAD-7 ≥ 10)	64 (36.99%)	60 (54.55%)	8.41	0.004 *
Moderate to severe depression (PHQ-9 ≥ 10)	73 (42.20%)	60 (54.54%)	4.12	0.042 *
Increased risk for disordered eating (EAT-26 ≥ 20)	47 (27.17%)	30 (27.27%)	<0.001	0.985

Data are presented as n (%). Statistical significance was determined using the Pearson Chi-square test, * *p* < 0.05.

**Table 5:** Comparison of mental health symptom severity and eating attitudes between patients with UC and CD.

Variable, Median (IQR)	CD (*n* = 173)	UC (*n* = 110)	U	*p*
Anxiety (GAD-7)	7.0 (5, 13.5)	10.5 (5.0, 14.0)	7920	0.017 *
Depression (PHQ-9)	8.0 (4.0, 14.0)	11.0 (5.0, 16.0)	8353	0.083
Eating disorders (EAT-26)	11.0 (4.0–21.0)	11.5 (3.0–21.0)	9209	0.648

Statistical significance was tested using the Mann–Whitney U test, * *p* < 0.05.

**Table 6:** Spearman’s correlations (ρ) among depressive symptoms (PHQ-9), anxiety (GAD-7) and eating attitudes (EAT-26) in CD and UC groups.

Spearman’s Correlation (*ρ*, *p*-Value)	PHQ-9 and EAT-26	GAD-7 and EAT-26	PHQ-9 and GAD-7
CD (*n* = 173)	0.317, <0.001	0.241, 0.001	0.719, <0.001
UC (*n* = 110)	0.278, 0.003	0.256, 0.007	0.662, <0.001

**Table 7:** Demographic characteristics of IBD patients, who participated in individual interviews.

Number of Interviewees (*N*)	Ν = 14
Disease (%)	
Crohn’s disease	9 (64%)
Ulcerative colitis	5 (36%)
Age (in years)	
Median (IQR)	44 (12.5)
Gender N (%)	
Men	7 (50%)
Women	7 (50%)
Area of residence N (%)	
Urban	13 (93%)
Rural	1 (7%)
Disease activity status N (%)	
Remission	9 (64%)
Flare-up	4 (29%)
Don’t know	1 (7%)
Previous surgery N (%)	
Yes	5 (36%)
No	9 (64%)
Type of treatment N (%)	
Biological agent	12 (86%)
Corticosteroids	3 (21%)
Immunosuppressants	4 (29%)
Antibiotics	1 (7%)
Other treatment	3 (21%)
Diagnosis N (%)	
11 years ago, or more	10 (71%)
6–10 years ago	1 (7%)
1–5 years ago	2 (14%)
7–12 months ago	1 (7%)

**Table 8:** Impact of various food categories on disease symptoms during relapse and remission periods.

**Relapse**		
**Positive**	**Negative**	**Neutral**
Rice Potato Chicken Fish Banana Apple Pear Hard cheese Boiled egg Avocado Boiled cauliflower Freshly squeezed pomegranate juice Feta cheese Kefir Ayran Chamomile tea Curcumin Magnesium supplement Enteral nutrition feeds	Legumes Foods containing lactose Foods containing gluten Red meat Coffee Nuts Vegetable dishes cooked in olive oil Fried foods Spices (e.g., pepper) Chocolate Pop corn Dried fruits Liver Soft drinks Onion Garlic Alcohol Seeds	Legumes, depending on the cooking method Toasted bread/Rusks Vegetables Fruits Fibers
**Remission**		
**Positive**	**Negative**	**Neutral**
Rice Potato Chicken Fish Boiled egg Boiled cauliflower Boiled carrots Boiled zucchini Soy milk Feta cheese Hard cheese	Eggplant Junk food Pepper Legumes Vegetable dishes cooked in olive oil Whole-grain products Foods containing lactose Fried foods Orange Boiled broccoli Onion Garlic Alcohol	Vegetables Fruits Foods containing gluten Legumes, depending on the cooking method

## Data Availability

The datasets used and/or analyzed during the current study are available from the corresponding author on reasonable request. The following email address will be requested: oandroutsos@uth.gr.

## Abbreviations

The following abbreviations are used in this manuscript:

CBT	Cognitive Behavioral Therapy
CD	Crohn’s Disease
EAT-26	Eating Attitudes Test-26
ECCO	European Crohn’s and Colitis Organisation
EEN	Exclusive Enteral Nutrition
GAD-7	Generalized Anxiety Disorder Scale-7
HPA	Hypothalamic–pituitary–adrenal
IBD	Inflammatory Bowel Disease
PHQ-9	Patient Health Questionnaire-9
UC	Ulcerative colitis
